# Dermatitis herpetiformis und sonstige Formen der Weizensensitivität

**DOI:** 10.1007/s00105-023-05243-1

**Published:** 2023-10-26

**Authors:** T. Malkovics, M. I. Joura, K. Koszorú, M. Sárdy

**Affiliations:** 1 Klinik für Dermatologie, Venerologie und Dermatoonkologie, Fakultät für Medizin, Semmelweis Universität, Mária u. 41, 1085 Budapest, Ungarn; 2 Universitätsklinik für Dermatologie, Medizinische Universität Wien, Wien, Österreich; 3 Klinik für Dermatologie und Allergologie, Klinikum der Universität München (LMU), München, Deutschland

**Keywords:** Transglutaminase, Reizdarmsyndrom, Nicht-Zöliakie-Weizensensibilität, Überempfindlichkeitsreaktion, Weizenabhängige anstrengungsinduzierte Anaphylaxie, Transglutaminase, Irritable bowel syndrome, Nonceliac gluten sensitivity, Hypersensitivity, Wheat-dependent exercise-induced anaphylaxis

## Abstract

**Hintergrund:**

Die Weizensensitivität ist ein Sammelbegriff für mehrere, v. a. gastrointestinale Erkrankungen, die im Rahmen einer Überempfindlichkeitsreaktion nach Weizenverzehr auftreten. Die meistens Reizdarmsyndrom-ähnlichen Symptome werden oft von Hautveränderungen begleitet. Die Weizensensitivität umfasst neben der Zöliakie und der Dermatitis herpetiformis (die bullöse kutane Manifestation der Zöliakie) auch die Nicht-Zöliakie-Glutensensitivität (NCGS), die allergische Nickel-Kontaktmukositis, die Weizenallergie, die Amylase-Trypsin-Inhibitor-Intoleranz sowie die FODMAP(fermentierbare Oligosaccharide, Disaccharide, Monosaccharide und Polyole)-Intoleranz.

**Ziel der Arbeit:**

Der Beitrag soll eine Übersicht über die klinischen, insbesondere dermatologischen und gastrointestinalen Eigenschaften der unterschiedlichen Weizensensitivitätsformen geben. Zudem werden die Diagnostik sowie mögliche Therapieformen erörtert.

**Material und Methoden:**

Es erfolgte eine selektive Literaturrecherche mit Evaluierung von durch die Autoren selbst erhobenen klinischen Daten.

**Ergebnisse:**

Die Hautveränderungen sind bei der Dermatitis herpetiformis sehr krankheitsspezifisch. Bei der Weizenallergie treten jedoch häufig Symptome auf, die auch für andere Erkrankungen typisch sind. Sonstige Formen der Weizensensitivität manifestieren sich primär mit gastrointestinalen Auffälligkeiten, aber auch extraintestinale Symptome kommen vor. Die Diagnostik ist oft komplex und bedarf disziplinübergreifender Zusammenarbeit mit Gastroenterologen. Die Therapie besteht aus einer weizen- bzw. glutenfreien Diät.

**Diskussion:**

Die Kenntnis unterschiedlicher und häufig auftretender dermatologischer Anzeichen einer Weizensensitivität ist von großer Bedeutung, da diese immer öfter im Zusammenhang mit einer gastrointestinalen Pathologie, Intoleranzreaktionen und Allergien diagnostiziert werden.

## Abstract (en)

**Background:**

Wheat sensitivity is a collective term for several, especially gastrointestinal, diseases that occur as part of a hypersensitivity reaction after wheat consumption. The symptoms, which are mostly similar to those of irritable bowel syndrome, are often accompanied by skin lesions. In addition to celiac disease and dermatitis herpetiformis, the cutaneous manifestation of celiac disease, wheat sensitivity also includes nonceliac gluten sensitivity (NCGS), allergic nickel contact mucositis, wheat allergy, amylase–trypsin inhibitor intolerance, and fermentable oligosaccharides, disaccharides, monosaccharides, and polyols (FODMAP) intolerance.

**Objectives:**

This review article aims to provide an overview of the clinical, especially dermatological and gastrointestinal features of the different forms of wheat sensitivity. Diagnosis and therapeutic management are also discussed.

**Materials and methods:**

A selective literature search was carried out with evaluation of our own clinical data.

**Results:**

The skin lesions in dermatitis herpetiformis are very disease-specific. In contrast, wheat allergy often shares signs and symptoms with many other diseases. Other forms of wheat sensitivity cause primarily gastrointestinal abnormalities, but extra-intestinal manifestations can also occur. Their diagnosis is often complex and requires cross-disciplinary collaboration with experts in gastroenterology. The therapy consists of a wheat- or gluten-free diet.

**Conclusions:**

Knowledge of the different and frequently occurring dermatological signs of wheat sensitivity is of great importance, because dermatological manifestations associated with gastrointestinal pathology, intolerance reactions, and allergies appear more and more frequently.

## Was versteht man unter Weizensensitivität?

Die Weizensensitivität ist ein häufig vorkommender Zustand, bei dem es sich um einen Sammelbegriff für unterschiedliche, v. a. gastrointestinale Erkrankungen handelt. Diese treten im Rahmen einer Überempfindlichkeitsreaktion nach dem Verzehr von Weizen auf. Obwohl viele Patient:innen mit Weizensensitivität zugleich auch glutenempfindlich sind, ist diese nicht mit einer Glutensensitivität (Zöliakie) gleichzusetzen. Zu den Symptomen einer Weizensensitivität zählen Reizdarmsyndrom-ähnliche Anzeichen. Da Weizen (v. a. Vollkornweizen) nicht nur Proteine, sondern auch viele andere Stoffe enthält, können die in diese Krankheitsgruppe gehörenden Entitäten je nachdem identifiziert werden, gegen welchen Bestandteil die Sensitivität besteht. Neben der Zöliakie und der Dermatitis herpetiformis (DH) gehören noch folgende Erkrankungen zu dieser Gruppe: Nicht-Zöliakie-Glutensensitivität (NCGS), Nickelallergie (allergische Kontaktmukositis), Weizenallergie, Amylase-Trypsin-Inhibitor-Intoleranz sowie FODMAP(fermentierbare Oligosaccharide, Disaccharide, Monosaccharide und Polyole)-Intoleranz [2]. Hautveränderungen treten bei der DH in Form von Bläschen an den Streckseiten und bei Weizenallergie oft als akute oder intermittierende Urtikaria bzw. Angioödem auf, während sie bei den anderen Krankheitsbildern eher selten vorkommen. Diese sind dennoch von Bedeutung, denn dermatologische Manifestationen im Zusammenhang mit gastrointestinaler Pathologie werden zunehmend häufiger beobachtet, teilweise vermutlich wegen der häufigeren präzisen Identifizierung von seltenen Darmerkrankungen.

## Dermatitis herpetiformis

Dermatitis herpetiformis (DH) ist eine chronische Autoimmunerkrankung, die mit intensivem Juckreiz und subepidermaler Blasenbildung einhergeht [6, 9]. Es handelt sich um die extraintestinale Form der glutensensitiven Enteropathie (Zöliakie). Sie wird als eine systemische, immunvermittelte Erkrankung definiert, deren Entwicklung sowohl durch genetische, immunologische als auch durch Umweltfaktoren beeinflusst wird. Die genetische Veranlagung ist identisch mit der der Zöliakie, und die Pathogenese weist viele Ähnlichkeiten auf [6, 9]. Während bei der Zöliakie das Hauptautoantigen die Transglutaminase 2 (TG2, Gewebstransglutaminase) darstellt, ist es bei der DH die Transglutaminase 3 (TG3, epidermale Transglutaminase) [17, 19]. Diese führt zur Bildung von TG3-Ig(Immunglobulin)A-Immunkomplexen an den Spitzen der papillären Dermis sowie in den Wänden der Blutgefäße und verursacht so die charakteristischen Hautsymptome. Die Epitophomologie und das Epitopspreading zwischen den Transglutaminasen können auch zu anderen durch Gluten ausgelösten Autoimmunerkrankungen, wie z. B. der Glutenataxie oder gluteninduzierten Neuropathien, führen [7]. Zudem besteht eine Neigung zu weiteren, glutenunabhängigen Autoimmunerkrankungen. In den eigens erhobenen klinischen Daten von 121 DH-Patient:innen kamen die Hashimoto-Thyreoiditis und die Psoriasis vulgaris am häufigsten vor (je 4 Patient:innen). Diabetes mellitus Typ I (2 Patienten), Morbus Basedow (1 Patient), Alopecia areata (1 Patientin), Raynaud-Phänomen (1 Patient), perniziöse Anämie (1 Patientin) und Hidradenitis suppurativa (1 Patient) wurden ebenso diagnostiziert (unveröffentlichte Daten).

### Symptome und Diagnostik der Dermatitis herpetiformis

**Figure 1:**
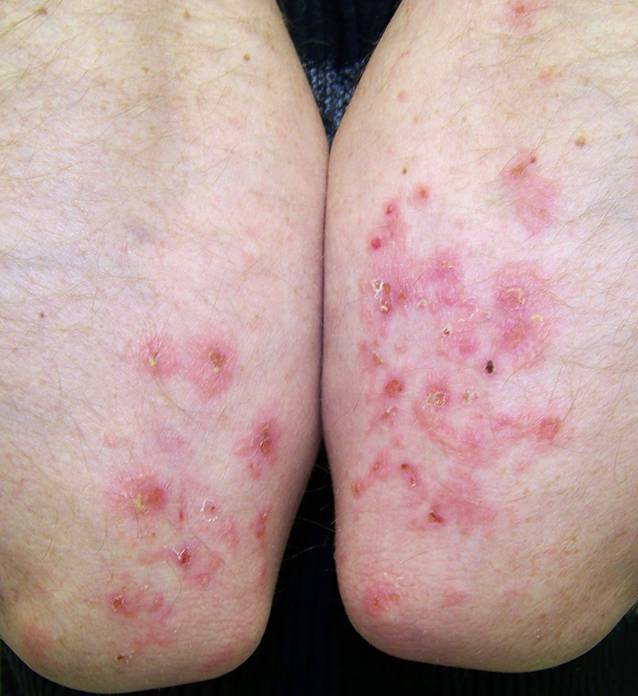

Das klinische Bild der DH ist hauptsächlich von polymorphen Läsionen geprägt, die in Druck oder Spannung ausgesetzten Hautpartien symmetrisch auftreten (z. B. Ellenbögen, Knievorderseiten, sakral und gluteal). Begleitet werden sie von brennendem Juckreiz, Exkoriationen sowie gruppiert angeordneten Vesikeln, Pusteln, exkoriierten Papeln und Plaques [6, 9]. Die Verteilung der Hautveränderungen ist charakteristischer als die eigentlichen Effloreszenzen (Abb. 1). Akrale Petechien und Purpura, die an den Fingern oder Zehen sichtbar sind, sind ebenfalls typisch [6, 9]. Gastrointestinale Symptome, die für Zöliakie charakteristisch sind (z. B. chronische Diarrhöen, Flatulenz, Obstipation, Bauchschmerzen, Gewichtsverlust usw.), treten bei 20–40 % der Patient:innen in milder Form auf [6, 10].

Das diagnostische Vorgehen soll auf der aktuellen europäischen Leitlinie basieren [6]. Die histologische Untersuchung allein ist zur Diagnosestellung nicht ausreichend, jedoch wichtig für die Differenzialdiagnostik. Die direkte Immunfluoreszenz (DIF) von intakter periläsionaler Haut ist der „Goldstandard“ in der Diagnostik der DH. Ein granuläres/fibrilläres IgA-Präzipitat ist an den Spitzen der dermalen Papillen und entlang der dermoepidermalen Junktionszone sichtbar [6, 9]. IgA-Autoantikörper gegen TG2 und TG3 können durch indirekte Immunfluoreszenz und ELISA („enzyme-linked immunosorbent assay“) nachgewiesen werden. Während die Sensitivität des serologischen Antikörpernachweises bei Zöliakie mehr als 95 % beträgt, liegt dieser Wert bei DH nur bei etwa 70 %. Daher ist die Durchführung der DIF zur Diagnosestellung in jedem Fall ratsam. Dies kann bei der Identifizierung von Hautveränderungen, die mit Zöliakie assoziiert sind, jedoch nicht mit einer DH in Verbindung stehen, helfen.

Die DIF ist der Goldstandard der Diagnostik von DH

Die serologischen Tests, die auf TG2 basieren, sind sowohl bei der Diagnosestellung als auch bei der Überwachung der glutenfreien Diät (quantitative TG2-basierte Tests) und bei der Differenzialdiagnostik von Bedeutung. Es gibt keine signifikanten Unterschiede in der Sensitivität und Spezifität zwischen dem semiquantitativen Endomysiumantikörpertest (indirekte Immunfluoreszenz) und dem quantitativen Anti-TG2-IgA-ELISA-Test. Daher wird empfohlen, nur eine dieser beiden Untersuchungen durchzuführen, da das Autoantigen identisch ist. Es wird nicht empfohlen, Antikörper gegen Gliadin oder deamidiertes Gliadin als Primärtests bei der diagnostischen Abklärung von DH zu verwenden [6].

Die Diagnose einer DH kann gestellt werden bei gleichzeitig bestehender zu DH passender kutaner Symptomatik und einer positiven DIF. DH verläuft oft (bei etwa 60 % der Patient:innen) ohne gastrointestinale Beschwerden. Dennoch ist die DH als kutane Form der Zöliakie zu verstehen, zumal sie mit einer meistens eher mild ausgeprägten glutensensitiven Enteropathie assoziiert ist. In der Jejunumbiopsie wird bei 75–80 % der Patient:innen histologisch eine Dünndarmatrophie festgestellt [6, 10].

### Therapie der Dermatitis herpetiformis

Die kausale Therapie besteht in einer lebenslang strikten Einhaltung einer glutenfreien Diät mit komplettem Verzicht auf glutenhaltige Produkte wie Weizen, Roggen, Dinkel und Gerste [6, 9]. Die glutenfreie Diät sollte erst nach Abschluss der vollständigen Diagnostik begonnen werden. Bis zum Erreichen einer kompletten Remission können Monate bis Jahre vergehen.

Die einzige kausale Behandlung ist eine lebenslange glutenfreie Diät

Bei schweren, ausgedehnten Hautsymptomen kann die glutenfreie Diät mit der Gabe von Dapson ergänzt werden. Der quälende Juckreiz und die Hautveränderungen bessern sich dadurch innerhalb weniger Tage. Es wird eine initiale Tagesdosis von 25–50 mg empfohlen, die bei Bedarf auf bis zu 150 mg erhöht werden darf [6]. Es sind v. a. in den ersten 3 Behandlungsmonaten Nebenwirkungen zu erwarten. Die häufigsten sind die dosisabhängige Methämoglobinämie sowie die hämolytische Anämie. Daher soll vor Therapiebeginn eine verminderte Aktivität der Glucose-6-Phosphat-Dehydrogenase (G6PD) ausgeschlossen werden, und es müssen anfangs wöchentliche Blutbildkontrollen durchgeführt werden, um ernsten Komplikationen vorzubeugen [6]. Dosisunabhängig können gelegentlich ein Arzneimittelexanthem (auch in Form eines DRESS[„drug-related eosinophilia with systemic symptoms“]-Syndroms), eine periphere Neuropathie oder eine Agranulozytose auftreten.

Die Diät sollte nicht durch eine Dapson-Therapie ersetzt werden. Eine systemische Behandlung sollte nur dann durchgeführt werden, wenn die Symptome intolerabel bzw. schwerwiegend sind, die Diät nicht akzeptiert wird oder die Krankheit auf eine korrekt durchgeführte Diät nicht ausreichend anspricht. Die Persistenz von kutanen oder abdominalen Symptomen ist trotz einer glutenfreien Diät möglich, allerdings ist dies oft ein Hinweis auf Diätfehler [10, 14]. Von einem Stoppen der glutenfreien Diät wird selbst nach mehreren Jahren Remission und Verschwinden der abgelagerten dermalen IgA-Autoantikörper abgeraten, da fast immer ein Rezidiv auftritt [11].

## Nicht-Zöliakie-Glutensensitivität

Die Nicht-Zöliakie-Glutensensitivität (NCGS oder Nicht-Zöliakie-Weizensensibilität [NCWS]) wurde erstmals in den 1970er-Jahren beschrieben, aber erst seit etwa 2010 steht sie im Fokus als eine mögliche neue, teilweise dermatologische Krankheitsentität v. a. bei jüngeren Patient:innen [1, 2]. Es wird derzeit noch diskutiert, ob diese tatsächlich eine eigenständige Erkrankung darstellt. Der Pathomechanismus ist unbekannt, keine genetischen Faktoren oder Biomarker wurden bislang identifiziert. Drei Symptomgruppen sind typisch für die NCGS [2]: Reizdarmsyndrom-ähnliche, neuropsychiatrische sowie systemische Symptome (Tab. 1). Die Erkrankung geht bei einigen Patient:innen mit stark juckenden, insbesondere an den Streckseiten der Extremitäten lokalisierten Hautveränderungen einher. Diese ähneln morphologisch einem Ekzem, einer Psoriasis oder einer Dermatitis herpetiformis, die zugleich die wichtigsten Differenzialdiagnosen darstellen [1]. Das histologische Bild ist diesen Differenzialdiagnosen entsprechend. Die DIF ist negativ oder zeigt nur eine granuläre Ablagerung von C3-Komplement entlang der Basalmembran, keine Immunglobulinablagerung. Serologisch kann man keine für Autoimmunerkrankungen typischen Auffälligkeiten finden, aber bei etwa 50 % der Patient:innen sind IgG-Anti-Gliadin-Antikörper positiv [1]. Es ist diagnostisch, dass die Symptome innerhalb von einem Monat auf eine standardisierte, doppelblinde, placebokontrollierte Glutenprovokation ansprechen [2, 3]. Andere, ähnliche Krankheiten wie Zöliakie oder die IgE-vermittelte Weizenallergie müssen ausgeschlossen werden.

Eine placebokontrollierte Glutenprovokation ist der Goldstandard der Diagnostik von NCGS

Therapeutisch ist ein strikter Verzicht auf alle glutenhaltigen Produkte notwendig. Die NCGS kann selbstlimitierend sein, daher sollte die Glutentoleranz im Verlauf von Zeit zu Zeit neu evaluiert werden [2, 3].

**Table 1:** 

Reizdarmsyndrom-ähnliche Symptome	Neuropsychiatrische Symptome	Systemische Symptome
Rezidivierende Bauchschmerzen	Konzentrationsstörung, Vergesslichkeit	Ermüdung
Diarrhö	Kopfschmerzen	Arthralgie
Obstipation	Angstzustände	Myalgie
Brechreiz	Depression	Fibromyalgie
Flatulenz	Verwirrung	Anämie
Rezidivierende aphthöse Stomatitis	Bein- oder Armparästhesie	
	Ataxie	

## Nickelallergie (allergische Kontaktmukositis)

Bestimmte Lebensmittel wie Hülsenfrüchte (z. B. Linsen, Erbsen, Bohnen), Tomaten, Nüsse, Kakao, Hafer und Weizenvollkorn enthalten eine ausreichende Menge an Nickel, um bei einer Nickelallergie Symptome an den Schleimhäuten auslösen zu können. Auch Nahrungsmittel, die in Alufolie aufbewahrt oder gebraten werden, können Nickel aufnehmen. Eine allergische Kontaktmukositis im Darm manifestiert sich mit Reizdarmsyndrom-ähnlichen Symptomen (Tab. 1). Die Diagnose kann mittels Nickel-Mundschleimhaut-Patchtest gestellt werden [15]. Nach 2‑stündiger Exposition führt dieser bei nickelempfindlichen Patient:innen zu spezifischen lokalen Veränderungen der Mundschleimhaut wie Ödemen und Hyperämie. Nach 24–48 h können zudem aphthöse bzw. vesikuläre Läsionen auftreten im Sinne von Typ-IV-Überempfindlichkeitsreaktionen.

In vielen Studien hat sich eine nickelarme Ernährung als wirksam, kostengünstig und nebenwirkungsarm erwiesen, abgesehen von einem hohen Obstipationsrisiko aufgrund einer unzureichenden Ballaststoffzufuhr.

## Weizenallergie

Die Weizenallergie ist eine IgE-vermittelte Typ-I-Allergie, die innerhalb von Minuten bis Stunden nach Weizenverzehr (Backwaren, Weizenbier, Sojasauce, Eiscreme, Kuchen, Nudeln usw.) Reizdarmsyndrom-ähnliche Symptome, Hautveränderungen und manchmal schwere Anaphylaxien verursacht. Anamnestisch kommt sie bei etwa 1,6 % der Bevölkerung in Europa vor, die Prävalenz der durch eine Provokationstestung bestätigten Weizenallergiker:innen liegt jedoch lediglich um 0,1 % [18]. Die Erstmanifestation kann bereits im Kleinkindesalter erfolgen [12], wobei allerdings die meisten Kinder eine Toleranz gegen Weizen bis zum Erwachsenenalter entwickeln.

Bei Weizenallergie im Kindesalter kommt es oft zur Toleranzentwicklung bis zum Erwachsenenalter

Erytheme, generalisierter Pruritus sine materia, chronisch rezidivierende Urtikaria und Quincke-Ödem sind häufige dermatologische Manifestationen [13]. Eine vorbestehende atopische Dermatitis kann sich ebenso durch Weizenverzehr verschlechtern.

Im Erwachsenenalter zählt Weizen zu den häufigsten Auslösern nahrungsmittelbedingter Anaphylaxien [4]. Von besonderer Relevanz ist hier die Weizen-abhängige anstrengungsinduzierte Anaphylaxie (WDEIA) mit Sensibilisierung gegen Omega-5-Gliadin, seltener gegen α/β/γ-Gliadine oder das Lipidtransferprotein Tri a 14 [5, 20]. Dabei handelt es sich um eine Sonderform der Weizensensitivität, bei der zwar Weizen generell vertragen wird, aber nach sportlicher Aktivität (oder im Zusammenhang mit anderen Kofaktoren wie NSAR[nichtsteroidale Antirheumatika]-Einnahme, Alkohol oder Infekten) allergische Reaktionen auftreten, wenn zuvor weizenhaltiges Essen verzehrt worden ist [16].

Aerogene Exposition gegenüber Weizenmehl kann v. a. zu Asthma bronchiale (Bäcker-Asthma), aber auch zu allergischen Hautreaktionen (z. B. Kontakturtikaria, Kontaktekzem) sowie Anaphylaxie führen.

Diagnostisch feststellen kann man Weizenallergien mithilfe eines positiven Pricktests und/oder durch den Nachweis spezifischer IgE-Antikörper gegen unterschiedliche Weizenproteine [5, 13]. Den letzten Beweis liefern bei Bedarf eine orale Provokationstestung, ggf. unter Berücksichtigung vermeintlicher Kofaktoren, und eine erfolgreiche Eliminationsdiät.

Manchmal findet man auch eine Immunantwort gegen Kontaminationen des Getreides wie Schimmel- oder Mehlmilbenproteine als Ursache der Beschwerden. Eine therapeutische Maßnahme stellt neben einer symptomatischen Behandlung mit Antihistaminika, systemischen Glukokortikoiden oder Adrenalin in der akuten Situation v. a. die langfristige weizen- oder glutenfreie Diät dar.

## Amylase-Trypsin-Inhibitor-Intoleranz

Unter den Weizenkornproteinen, die eine immunologische oder allergische Reaktion auslösen können, finden sich neben den Vorgenannten auch Amylaseinhibitoren (Amylase baut die Weizenstärke ab) sowie Trypsin- und Serinproteaseinhibitoren. Sie kommen nicht nur im Weizen, sondern auch in anderen Getreidearten vor. Die Weizen-Amylase- und -Trypsin-Inhibitoren (ATIs) sind eine Familie von Resistenzmolekülen, die für die Abwehr gegen Schädlinge und Parasiten verantwortlich sind [2].

Einige Mitglieder der ATI-Familie wurden als starke Auslöser angeborener Immunantworten in Makrophagen, Monozyten und dendritischen Zellen identifiziert. Es wurde nachgewiesen, dass die Getreide-ATIs dadurch zur Freisetzung proinflammatorischer Zytokine beitragen [8]. Dieser Effekt kann bei allen entzündlichen Darmerkrankungen die Krankheitsaktivität erhöhen und dadurch ihren Verlauf verschlimmern. Eine ATI-Eliminationsdiät (getreidefreie Diät) kann daher bei vielen Darmerkrankungen (Zöliakie, Colitis ulcerosa, Weizenallergie) effektiv sein.

## FODMAP-Intoleranz

FODMAP ist die englische Abkürzung für bestimmte verdaubare Kohlenhydrate in Lebensmitteln: „Fermentable Oligo‑, Di‑, Monosaccharides, and Polyols“. Zu FODMAP-reichen Lebensmitteln zählen Früchte (Apfel, Birne, Pflaume, Pfirsich), Gemüse (Zwiebel, Knoblauch, Salat, Erbsen) und Getreide (Weizen, Roggen, Hafer). FODMAPs (z. B. Fruktose, Saccharose, Laktose und Sorbitol) können schlecht absorbiert, jedoch schnell fermentiert werden, daher sind sie im Darm osmotisch aktiv [2]. Die Symptome entstehen durch Fermentierung, die zu Gasproduktion, osmotischer Diarrhö mit Bauchschmerzen, Veränderungen der intestinalen Mikrobiota und erhöhter Darmwandpermeabilität führt. Die FODMAP-Intoleranz kann unspezifisch auch mit Hautmanifestationen, wie z. B. chronisch rezidivierender Urtikaria, einhergehen. Bei Verdacht auf eine FODMAP-Intoleranz kann eine probatorische FODMAP-arme Diät über mindestens 4 Wochen empfohlen werden, die bei Beschwerdebesserung fortgeführt werden sollte.

## Sonstige möglicherweise mit Weizensensitivität assoziierten Erkrankungen

Gluten kann einige seltene Fälle der folgenden Erkrankungen verursachen: Psoriasis, Ataxie, Psychose mit Halluzinationen, Depression, Schizophrenie, Autismus.

## Fazit für die Praxis

- Es gibt eine Reihe von Weizenkornkomponenten, die eine immunologische oder allergische Reaktion auslösen können.
- Dermatitis herpetiformis (DH) zeigt stark juckende, polymorphe Hautveränderungen an den Streckseiten der Extremitäten bzw. sakral und gluteal. Die Diagnose wird aufgrund der klinischen Symptome, spezifischer serologischer Tests und der direkten Immunfluoreszenz (DIF) als Goldstandard gestellt.
- Hauptbestandteil der Therapie der DH ist die lebenslange glutenfreie Diät.
- Weizensensitivität kann sich mit unterschiedlichen chronisch rezidivierenden, inflammatorischen Hautveränderungen manifestieren. Ein breites Spektrum an Symptomen rechtfertigt die Diagnostik in Richtung Weizensensitivität.
- Die Diagnostik der Weizensensitivität ist komplex und bedarf einer engen Zusammenarbeit mit Gastroenterolog:innen.
- Weizensensitive Patient:innen können von einer getreidefreien Diät stark profitieren. Daher ist das Ausprobieren dieser Diät bei chronisch rezidivierenden, therapieresistenten, inflammatorischen Dermatosen kein bloßer Modetrend.

## Abkürzungen

ATIs: Weizen-Amylase- und -Trypsin-Inhibitoren
DH: Dermatitis herpetiformis
DIF: Direkte Immunfluoreszenz
DRESS: Drug-related eosinophilia with systemic symptoms
ELISA: Enzyme-linked immunosorbent assay
FODMAP: Fermentierbare Oligosaccharide, Disaccharide, Monosaccharide und Polyole
G6PD: Glucose-6-Phosphat-Dehydrogenase
Ig: Immunglobulin
NCGS: Nicht-Zöliakie-Glutensensitivität
NCWS: Nicht-Zöliakie-Weizensensibilität
NSAR: Nichtsteroidale Antirheumatika
TG2, TG3: Transglutaminase 2 bzw. 3
WDEIA: Wheat-dependent exercise-induced anaphylaxis
